# Nutrients, Nutraceuticals, and Xenobiotics Affecting Renal Health

**DOI:** 10.3390/nu10070808

**Published:** 2018-06-23

**Authors:** Carmela Cosola, Alice Sabatino, Ighli di Bari, Enrico Fiaccadori, Loreto Gesualdo

**Affiliations:** 1 Department of Emergency and Organ Transplantation—Nephrology, Dialysis and Transplantation Unit, University of Bari Aldo Moro, 70124 Bari, Italy; ighli87@gmail.com (I.d.B.); loreto.gesualdo@uniba.it (L.G.); 2 Department of Medicine and Surgery, Parma University Medical School, 43126 Parma, Italy; alice.sabatino86@gmail.com (A.S.); enrico.fiaccadori@unipr.it (E.F.)

**Keywords:** CKD, renal function, nutrients, nutraceuticals, xenobiotics, inflammation, functional nutrition

## Abstract

Chronic kidney disease (CKD) affects 8–16% of the population worldwide. In developed countries, the most important risk factors for CKD are diabetes, hypertension, and obesity, calling into question the importance of educating and acting on lifestyles and nutrition. A balanced diet and supplementation can indeed support the maintenance of a general health status, including preservation of renal function, and can help to manage and curb the main risk factors for renal damage. While the concept of protein and salt restriction in nephrology is historically acknowledged, the role of some nutrients in renal health and the importance of nutrition as a preventative measure for renal care are less known. In this narrative review, we provide an overview of the demonstrated and potential actions of some selected nutrients, nutraceuticals, and xenobiotics on renal health and function. The direct and indirect effects of fiber, protein, fatty acids, curcumin, steviol glycosides, green tea, coffee, nitrates, nitrites, and alcohol on kidney health are reviewed here. In view of functional and personalized nutrition, understanding the renal and systemic effects of dietary components is essential since many chronic conditions, including CKD, are related to systemic dysfunctions such as chronic low-grade inflammation.

## 1. Introduction

Among the environmental factors that affect human health, nutrition is of utmost importance, since dietary habits heavily influence the incidence and progression of a variety of pathologies, particularly non-communicable diseases (NCDs). Chronic kidney disease (CKD) is an NCD affecting 8–16% of the population worldwide, with diabetes, hypertension, and obesity being the most important risk factors for its occurrence in developed countries [1]. 

The importance of nutrition in nephrology, with a special focus on protein and salt restriction, has long been recognized as crucial for the management of CKD patients, along with pharmacological therapy to slow down disease progression and correct the signs and symptoms of uremia [2]. On the contrary, notions about the role of nutrition as a measure to prevent renal disease are less discussed. 

A balanced diet and, if necessary, supplementation can indeed support the maintenance of general health status and the preservation of renal function, and can help to manage and curb the main risk factors for renal damage: diabetes, hypertension, and obesity. In this respect, protein [3] and fiber [4,5,6] intake is a fundamental tool in the dietary control of these risk factors. Some evidence does exist pointing to the importance of gut microbiota balance in the prevention of renal function decline [7], with fiber playing a primary role in the modulating the microbial metabolism. In a systemic and functional view of nutrition, controlling low-grade chronic inflammation is crucial for renal damage prevention. In this regard, functional molecules and nutrients such as fiber and fatty acids, and plant-derived nutraceuticals such as curcumin and steviol glycosides, play a key role, either influencing pro- and anti-inflammatory pathways or acting at the gut mucosal level [5,8,9]. Gut permeability is indeed pivotal in the etiology and progression of immune disorders and in eliciting chronic inflammation, since exposure of gut-associated lymphoid tissue (GALT) to luminal antigens is one of the main triggers of these conditions [10]. Different dietary components can modulate gut barrier integrity both directly and indirectly, and there is evidence that curcumin and fiber have positive effects in this regard. The beneficial effects of plant-based diets in hypertension are well known [11], along with those of sodium reduction [12]. We discuss here the less-known role of dietary fiber, nitrates, nitrites, and stevia in endothelial function and blood pressure (BP) control [13,14]. Moreover, we report the effects of tea and coffee polyphenols, as well as those of bifaceted alcohol, on renal health. 

In this narrative review, we decided to focus on some pivotal nutrients, and on some less-studied nutraceuticals and xenobiotics, reporting literature evidence of their beneficial or detrimental action, and in some cases offering potential treatment perspectives. We report here the current scientific evidence on fiber, protein, fatty acids, curcumin, stevia, green tea, coffee, nitrates, nitrites, and alcohol, and on their direct and indirect effects on renal health, both in CKD and non-CKD individuals.

## 2. Fiber

From the human nutrition point of view, dietary fiber is not strictly classified as a “nutrient” since it is technically indigestible by the human enzymatic panel, some of it instead being metabolized by intestinal microbial enzymes. For this reason, and given the paramount importance given to the gut microbiota in human health research, dietary fiber intake is attracting more and more attention [15]. Fiber is traditionally classified into insoluble and soluble, the first being responsible for stool weight and laxation and the second having serum lipid-lowering effects. However, this rough categorization has recently been challenged [4]. Even so, when it comes to considering the health benefits of intestinal microbial fermentation, soluble fiber is of particular interest since it includes the category of prebiotic fiber, characterized by the ability to escape digestion, reach the distal tract of the gastrointestinal tube intact, be selectively fermented by probiotic bacteria, and induce the growth of the latter [4].

Given the crucial role of microbial dysbiosis in CKD progression and comorbidities, our group and other investigators have recently highlighted the importance of adequate fiber intake in the dietary management of CKD [16,17,18,19,20,21,22]. Indeed, fiber intake reduces proteolytic putrefaction and increases saccharolytic fermentation [9,14,17], potentially counteracting the putrefactive dysbiosis that accompanies the decline in renal function [19,23,24].

On these grounds, we have assumed the potential preventative action of fiber on renal function decline, although this cause–effect relationship can only be guessed at at the moment as, to the best of our knowledge, we still lack experimental confirmation from large-scale observational studies.

Importantly, a recent work has demonstrated the crucial role of intestinal wellness as a protective factor for renal health, underlining the close connection between the gut and the kidneys [21]. The authors of this study found that constipation status and severity were correlated with an increased risk of incident CKD, end-stage renal disease (ESRD), and progressive decline of estimated glomerular filtration rate (eGFR) in a large cohort of 3,504,732 U.S. veterans [21]. The reasons underlying this association are unknown, but likely related to uremic toxin retention, increased intestinal barrier permeability, and colonic and systemic inflammation. 

**Figure 1:**
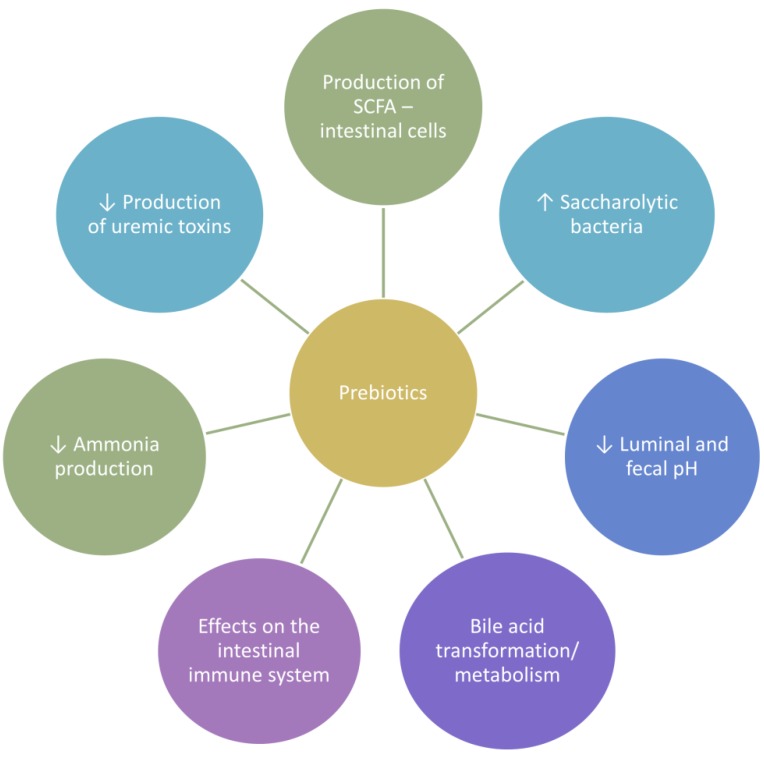
Beneficial effects of prebiotic fiber on the intestine. SCFA: short-chain fatty acids.

Indeed, fiber is fundamental for intestinal and general wellness (Figure 1). It serves as nutrient for the saccharolytic microbiota, and plays a role in decreasing local and systemic inflammation by ameliorating gut barrier integrity through the release of short-chain fatty acids (SCFA) [5,9]. In addition, it guarantees optimal bowel transit, favoring the enteric excretion of human and bacterial metabolites and preventing their systemic accumulation [20,21]. Moreover, there are some hints suggesting that dysbiotic microbiota could represent a risk factor for CKD occurrence in genetically or epigenetically predisposed subjects [7,25]. The resulting reduction of uremic toxin production, induction of SCFA colonic release, decreased intestinal inflammation, and restored intestinal barrier are all factors potentially contributing to renal health by reducing the risk of renal function decline. 

Finally, fiber is an important ally in the control of metabolic and cardiovascular (CV) conditions known to be risk factors for CKD: obesity, diabetes, and hypertension [4,5,6]. An adequate fiber intake can indeed modulate human metabolism thanks to its action on glycemic index reduction [26], satiety induction, cholesterol absorption, and BP reduction [4]. Dietary fiber content in diet is also effective in supporting weight loss and glycemic control, probably not only by the mechanisms of satiation, delayed gastric emptying, and decreased absorption of nutrients [27,28], but also by modulating the gut endocrine and metabolic orchestra [5,27].

Among the CKD risk factors, the action of fiber on BP has not been yet extensively studied. While the indirect protective effect of fiber at CV level, through the control of blood glucose and lipid levels, has been well demonstrated [6,13], some recent studies also suggest a direct action of fiber types such as beta-glucans, psyllium, lupin kernel, soluble cocoa, and grape fiber on endothelial function and hypertension [13,14].

## 3. Protein

In the context of CKD, the positive clinical effects of a low-protein diet (LPD) are not only related to the control of uremic symptoms, reduction in proteinuria, and hyperfiltration, but also to the related reduction of sodium, inorganic acids, and phosphorus content. Favorable renal outcomes have been reported with LPD. In the early stages of CKD, a normalization of the protein intake based on the current recommendations for the general population (0.8 g/Kg/day) is advised. With the worsening of renal function (CKD stages 3 and 4), more restrictive diets are necessary (0.6–0.7 g/Kg/day) (Table 1). 

Some experts advocate the use of a very low protein diet (VLPD) supplemented with ketoacids in CKD stage 5 to delay dialysis initiation, or as a way to keep the elderly free from dialysis. Starting dialysis in patients older than 75 is indeed related to an increased risk of mortality in the first year following initiation of hemodialysis (HD), and to the worsening of physical function and quality of life [30]. Available data show that in patients with moderate to advanced CKD, VLPD supplemented with ketoacids improves several metabolic abnormalities, including hyperphosphatemia, metabolic acidosis, hyper-parathyroidism, dyslipidaemia, protein carbamylation, and high urea levels [31,32,33,34]. VLPD also contributes to the control of proteinuria, BP, and hemoglobin [35,36,37,38], without compromising nutritional status [39,40]. Despite inconclusive data on the role of LPDs in preventing or slowing down additional loss of kidney function, the main role of this diet in the more advanced stages of CKD is to control CKD-related metabolic abnormalities. Even a slight reduction in protein intake of 0.2 g/kg/day may significantly improve the uremic state, metabolic acidosis, and hyperphosphatemia [33,41]. 

Given the LPD protective role in CKD, the hypothesis is that unrestricted protein intake in the presence of a decreased number of functioning nephrons can lead to increased glomerular capillary pressure and result in single-nephron hyperfiltration [42]. These hemodynamic changes could contribute to glomerulosclerosis and further reduction in the number of functioning nephrons. In fact, a 32% relative risk reduction in renal death in favor of a LPD over a higher protein intake was identified in a meta-analysis of 2000 patients [2]. A 10 g increase in protein intake in otherwise healthy females with mildly reduced renal function (eGFR > 55 but <80 mL/min per 1.73 m^2^) was related to a significant reduction in eGFR over an 11-year period compared to women with normal renal function [43]. High protein intake was also associated with a worsened GFR compared with moderate and low protein intake in non-dialyzed stage 3 to 5 CKD patients [44]. When initiating a LPD, CKD patients should receive professional nutritional counseling, since unsupervised protein restriction may lead to protein energy wasting (PEW) if calorie requirements remain unmet, thus leading to poor outcomes in terms of future morbid events, progression of renal disease, and mortality [45]. Nevertheless, the problem of being on a LPD and at the same time meeting calorie requirements can be quite common in patients with CKD, even when professional nutritional counseling is available. Supplementation with essential amino acids allows for the intake of lower quality protein, better palatability of the diet, and a broader choice of foods [39]. LPDs can also be supplemented with special protein-free foods. Today these products are readily available as pasta, cookies, bread, and flour but also as precooked soups and desserts, accounting for an invaluable resource for optimal low-protein dietary management of CKD with higher energy intake, no additional phosphate and protein supply, and a lower sodium content.

Unlike CKD patients, healthy people are advised to ingest a recommended daily allowance (RDA) of 0.8 g of proteins/kg body weight. This amount, which has been obtained by theoretical studies based on measurements of nitrogen urinary waste, assumed to be proportional to the body nitrogen turnover, indicates the minimum quantity necessary to cover the basal daily protein need of 97.5% of healthy people. Protein exerts different beneficial functions for human health. First, they guarantee an optimal structural turnover, both at an extra- and an intracellular level. Second, they are crucial for the functioning of the immune system. Third, protein is particularly required in some special life phases and conditions: pregnancy and infancy, the elderly, and intensive physical activity. They also become critical in the context of low-calorie diets aimed at weight loss, as they prevent lean mass waste [3].

The prevalent notion in the field of kidney disease that a strict control of protein intake is always required has led to the misleading idea that even in healthy people an excess of protein intake over the RDA could be harmful for kidney health, leading to increased CKD risk [46]. To date, no long-term study has confirmed the association between high protein intake and increased risk of CKD in the healthy population [46,47]. This has been confirmed by a research conducted on a population of 1624 women belonging to the Nurses’ Health Study, demonstrating that high protein intake was not associated with renal function decline in women with normal renal function, instead being associated with a decline in renal function only in women with pre-existing mild renal insufficiency [43]. The hyperfiltration observed following high-protein diets seems to be linked more to an adaptive mechanism than to direct kidney damage [46,47], even if the long-term effects of a chronically elevated protein intake cannot be excluded [48]. On the contrary, insufficient protein intake leads to a reduced kidney volume and filtration rate [49,50].

In summary, while CKD patients should be compliant with protein restrictions, in the general population a balance should be struck between risks and benefits. Obesity and its comorbidities such as hypertension, insulin resistance, and diabetes currently represent the main risk factors for CKD. Diets with a protein intake that exceeds the RDA still represent a valuable tool for weight control and should therefore be used in order to achieve or maintain the weight loss goal, or to prevent malnutrition in the elderly [3]. In the presence of a normal renal function, there is no evidence against the adoption of high-protein diets for specific health purposes. These diets should be customized according to the clinical and nutritional pictures of the individual patients and supervised by a nutritional specialist. 

## 4. Fatty Acids 

Fatty acids (FA) are important lipid components, mainly represented by triglycerides and phospholipids, which are derived from food or can be produced endogenously; they play an important role in chronic degenerative diseases with an inflammatory component [51]. In fact, circulating FA levels are considered an index of endogenous production, dietary intake, and pathophysiological conditions. Long-chain polyunsaturated FAs (LC-PUFA) cannot be produced endogenously in enough quantity, and thus are considered essential FAs. Essential FAs are known as omega-6 and omega-3 FAs and are widely distributed in plant oils. Longer-chain fatty acids derived from the parental omega-3 FA alpha-linolenic acid (ALA) are partially produced endogenously, and are considered essential too: they are eicosapentaenoic acid (EPA) and docosahexaenoic acid (DHA), widely available in fatty fish. A great bulk of data on the association between PUFA metabolism and CV risk factors in the general population is available [52,53]. 

Significant modifications in the FA profile of circulating lipids have been observed in patients with CKD and nephrotic syndrome [54]. However, whether this condition is a consequence or a cause of the renal dysfunction is still a matter of debate. Only a few studies have investigated FA levels in CKD patients in comparison with healthy subjects. Taken together, the literature studies on patients with nephrotic syndrome show a reduction in the stearic acid levels (a type of saturated fatty acid, SFA), as well as a reduction in omega-3 and a change in the enzymatic indicators of FA metabolism and synthesis [55,56,57]. As to CKD patients, the studies performed before any FA supplementation found lower levels of omega-3 FA, DHA, and SFA and increased levels of omega-6 FAs and oleic acid (a monounsaturated fatty acid, MUFA) [58,59,60]. The presence of proteinuria could explain more than 60% of the altered FA levels in nephropathy patients, since urinary phospholipid levels correlate directly with proteinuria [57]. In addition, the presence of hypoalbuminemia is likely to contribute to the change in FA profiles [61]. Finally, the increased levels of MUFA and reduced levels of PUFA found in CKD patients could be a result of an increased oxidative stress, which affects the structure of PUFAs more than that of MUFAs and SFAs [62,63]. 

On the other hand, FAs imbalance may be not only a consequence of the kidney disease but also a factor influencing kidney function. The association between PUFA levels and the progression of CKD has also been documented, with increased levels of omega-6 PUFA arachidonic acid (AA) being responsible for the upregulation of mediators of the renal fibrotic process [64,65,66]. In addition, a change in PUFA levels can increase the expression of angiopoietin like-4 (Angpt-4), which encodes a glycosylated, secreted protein containing a C-terminal fibrinogen domain that can act as an apoptosis survival factor for vascular endothelial cells, and could have a key role in nephritic syndrome [67]. As to the development of comorbidities, an increase in the omega-3/omega-6 ratio, represented by DHA/AA and EPA/AA, has been shown to be protective against CV events in CKD patients [68], while an increase in the AA/n-6 dihomo-γ-linoleic acid (DGLA) ratio in HD patients has been found to be an independent predictor of poor clinical outcomes [69]. In general, higher levels of mead acid, an indicator of essential fatty acid deficiency, have been associated with increased levels of the inflammatory cytokine IL-6 and increased mortality [70]. 

In kidney disease, the positive or negative roles of FAs mainly depend on the effects of their metabolites [71,72] on the modulation of the inflammatory status and tissue fibrosis [73]. The epoxyeicosatrienoic (EET) acids derived from the AA are a family of metabolites with important effects on the kidney [54]. They inhibit Na channels at the cortical collecting duct [74], maintain the glomerular permeability barrier to albumin [75], and play a protective role against glomerular damage [76]. Like EETs, 20-hydroxyeicosatetraenoic (20-HETE) acid, another metabolite derived from AA, plays an important role in maintaining the integrity of the glomerular barrier to albumin [75], and has been demonstrated to have a protective role on ischemia–reperfusion (IR) injury [77]. Omega-3 FA metabolites also have important roles in the kidney, mainly related to counteracting inflammation. Resolvins derive from DHA and facilitate the resolving phase of acute inflammation [78], and the metabolite 4-hydroxy hexenal increases the expression of genes with antioxidant and anti-inflammatory properties [79,80,81,82].

There are many studies investigating the role of omega-3 PUFA supplementation in CKD, and their results regarding its protective effect on renal function are controversial [83,84,85,86,87,88] (Table 2). The heterogeneity found in such trials can be explained by differences in basal EPA and DHA status, not always measured in every trial, type of omega-3 FA used (parental ALA, EPA or DHA), route of the supplementation (oral/parenteral), dose and non-linear dose response effects, duration of the intervention, and differences in the levels of mediators [89]. All these factors must be taken into account in clinical practice when considering supplying CKD patients with omega-3. To this end, it would be advisable to perform a thorough determination of the FA status of patients in order to identify the right dose and kind of FA supplementation.

## 5. Nutraceuticals and Xenobiotics 

### 5.1. Curcumin

Curcumin is a biologically active polyphenolic compound found in turmeric, a spice derived from the rhizomes of the plant *Curcuma longa*, known for its pharmacologic, anti-inflammatory, and anti-cancer properties [90,91]. 

In recent years, different scientific studies have highlighted the nutraceutical properties of this molecule at a systemic and also a renal level. In the field of renal diseases, some in vivo and human studies suggest new therapeutic perspectives for curcumin use in CKD [92,93], acute kidney injury (AKI) [94], transplantation [95,96], and renal cell carcinoma [91]. The results of these research efforts need to be validated by RCTs. 

In a recent in vivo study, oral administration of curcumin in mice models of CKD induced several beneficial effects: amelioration of cardiac function, decrease of BP and ROS production, and improvement of mitochondrial integrity and functionality. While renal function seemed to be unaffected by curcumin administration, reduced gelatinolytic activity of metalloproteinases and decreased expression of MMP-2, an extracellular matrix remodeling enzyme, were observed [92]. Collectively, curcumin appeared to reduce cardiac remodeling, mitochondrial dysfunction, and cell death mediated by oxidative stress. The antioxidant effect of curcumin has recently been confirmed in a double-blind RCT carried out on diabetic and nondiabetic proteinuric CKD patients [93].

An important role of curcumin has also been shown in relation to the IR injury-induced AKI. Following IR induction in an animal model, lower levels of serum creatinine and urea were observed in the group pre-treated with curcumin as compared to the control group. At the molecular level, positive effects on tubular epithelial cells have been observed: curcumin prevented their apoptosis by increasing the expression of APPL1 and inhibiting protein-kinase B (AKT) phosphorylation, a pivotal pathway for the pathogenesis of IR-induced AKI [94]. 

Biological effects of curcumin have also been reported in the context of clear renal cell carcinoma (ccRCC), a tumor notoriously resistant to chemotherapeutic treatments. In an in vitro study curcumin showed a double effect on RCC cells, promoting their viability at low doses and inducing their autophagy at high doses [91]. This observation underlines the importance of carrying out accurate dose–effect in vitro and in vivo studies, and ultimately human trials, in order to determine the optimal pharmacological dose of nutraceutical compounds. 

Studies on curcumin activity have been performed in renal transplantation, too. A RCT demonstrated that curcumin is effective in the functional recovery of a kidney transplanted from a cadaver, a condition in which the delayed graft function is very common, implying the acute rejection of the organ. Forty-three HD-dependent cadaveric kidney recipients received a curcumin-based supplement for one month after surgery in a randomized scheme. Results showed an improved early graft function in the curcumin-treated group, along with a reduced incidence of acute rejection and neurotoxicity [95]. 

Apart from potential therapeutic applications, curcumin could also be important in terms of prevention of renal function decline, thanks to its multifaceted actions. As already reported for CKD animal model and human studies [92,93], this molecule exhibits antioxidant properties, potentially useful in a preventative setting. In this regard, in a very recent animal study curcumin has been shown to be able to prevent early damage induced by 5/6 nephrectomy, not only ameliorating total antioxidant capacity, but also improving renal blood flow and acting on mitochondrial function and bioenergetics [97].

Perhaps the most interesting action of curcumin occurs at the intestinal level, resulting in systemic effects. Indeed, there are some reports showing the capacity of curcumin to preserve the intestinal barrier integrity, thus reducing systemic inflammation. Among the various stimuli known to alter gut permeability and exposing gut-associated lymphoid tissue to mucosal antigens is intestinal lumen lipopolysaccharide (LPS). LPS, a component of the Gram negative bacterial wall, is able to promote the downregulation of tight junction proteins ZO-1 and claudin, increasing intestinal permeability to LPS and other metabolites [98]. This results in a cascade of inflammatory events, including increased secretion of pro-inflammatory cytokines and chemokines, activation of macrophages, and their infiltration within the renal tissues and the arterial walls, all predisposing to the development of CKD and atherosclerosis [99,100,101]. Intestinal alkaline phosphatase (IAP) has a significant role in preserving mucosal barrier integrity. It is a membrane enzyme expressed by the intestinal epithelium, having the important role of detoxifying luminal LPS through the removal of one of the two phosphate groups from the lipid A [98,102,103]. The reduction of IAP (occurring in Western-style diets as well in CKD) results in increased active LPS, which in turn causes the downregulation of ZO-1 and claudin. Oral intake of curcumin promotes increased IAP activity, reversing the downregulation of tight junction proteins [8]. 

### 5.2. Stevia

Stevia is a food additive extracted from the leaves of *Stevia rebaudiana*. Its bioactive elements are steviol glycosides, mainly rebaudioside A and stevioside [104]. With a sweetening index much higher than that of sucrose [105], stevia is not fermentable, is stable at different pH values and heat-resistant and, importantly, has no caloric value [106]. The beneficial properties of stevia have been evaluated in some animal studies over the past 20 years. The positive effects of stevia on the kidneys have been known since the early 1990s. In an in vivo study, Melis dispensed 2.67 g of dry stevia leaves/day for 30 days to normal and hypertensive rats, showing different beneficial effects such as BP reduction, amelioration of GFR, and increase in renal plasma flow and sodium excretion [107,108]. 

In a follow-up study, the effects of polyphenols and fiber extracted from stevia leaves on streptozotocin-induced diabetic rats were studied. In comparison with the control group, rats administered with stevia powder and polyphenols extracts showed a decrease in blood glucose levels, an improvement in liver function evidenced by the modulation of AST and ALT blood levels, an increase in antioxidant enzymes levels, and a decrease in hepatic malondialdehyde (MDA) (an indicator of hepatic damage and a marker of oxidative stress) concentration. Importantly, an improvement of the GFR after the renal damage was observed in the treated group [109].

An interesting aspect related to the effect of stevia has been found in autosomal dominant polycystic kidney disease (ADPKD). Stevioside is metabolized by intestinal bacteria to steviol, which is readily absorbed at the intestinal level, also reaching the kidney. In an animal model of ADPKD, some authors have described a key role for stevioside and steviol in delaying cystogenesis and improving renal function, through the activation of AMP-activated protein kinase, in its turn inhibiting CFTR and mTOR/S6K protein expression, all involved in renal epithelial cell proliferation [110]. Moreover, steviol allowed a decrease in kidney weight and size, and cyst index and area, compared with the control group. Furthermore, stevioside treatment also promoted similar effects such as reduced kidney weight and decreased cyst index, but, unlike steviol, an improvement in serum blood urea nitrogen and creatinine was not observed [111]. Furthermore, steviol enhanced the expression of lysosomal enzyme marker (LAMP2), indicating the lysosomal degradation of CFTR (playing an important role in fluid accumulation in cysts) and of B-catenin (important in the cell proliferation pathway). Collectively, the modulation of these pathways results in a slowdown of the cysts’ progression [112].

Aquaporin 2 (AQP2) is involved in fluid secretion, promoting cyst enlargement in polycystic kidney disease (PKD). Noitem et al. found that steviol remarkably inhibited cyst growth in vitro by decreasing AQP2 expression in mouse renal cystic epithelial cells [113]. 

Given steviol glycosides’ beneficial properties and potential therapeutic applications, it would be appropriate to test these functional molecules in pilot studies on CKD patients as well as in pharmacological studies meant to develop new innovative drugs by harnessing their chemical structure.

### 5.3. Green Tea and Coffee

Green tea contains polyphenolic compounds (flavonoids) displaying antioxidant properties, known as catechins. These compounds have shown anti-oxidative, anti-inflammatory, and anti-carcinogenic activity [114,115,116]. There is evidence that increased antioxidant status lowers oxidative damage to DNA, thus enhancing protection against cancer [114,117]. In addition, epidemiological data have indicated a lower incidence of cancer in subjects with higher intake of green tea [118,119,120]. Since oxidative stress and inflammation contribute to the development and progression of renal diseases, one could speculate that frequent consumption of green tea or green tea extracts could play a protective role on renal function. In a recent animal study [121], researchers investigated the renoprotective role of epigallocatechin-3-gallate (EGCG), the most abundant and active polyphenol present in green tea [117], in models of unilateral ureteral obstruction (UUO). The main findings of this study were that EGCG administration at a dose of 50 mg/Kg/day significantly improved renal function and increased the weight of the obstructed kidney in mice. In addition, EGCG counteracted the UUO effects by normalizing the antioxidant and inflammatory activity [121]. In another very recent study, catechin administration at different dosages after cadmium exposure significantly attenuated the nephrotoxic effects of cadmium exposure by reducing oxidative stress and inflammation and by protecting the renal mitochondrial structure and function [122]. A protective role of EGCG in the development of diabetic nephropathy in mice has also been reported [123,124,125,126,127], inducing the upregulation of the nuclear factor erythroid 2-related factor 2 (NRF2), which plays a key role in cellular defense against diabetes-induced oxidative stress [127]. In a double-blind RCT, supplementation with green tea polyphenols containing 800 mg of EGCG was able to reduce albuminuria in patients with diabetic nephropathy receiving the maximum recommended dose of renin–angiotensin system (RAS) inhibition. The mechanisms involved a reduction in the podocyte apoptosis mediated by WNT pathway activation [128]. Additionally, recent findings show that antioxidant supplementation inhibits the progression of atherosclerosis and inflammation [129]. Catechins can inhibit pro-inflammatory and pro-apoptotic oxidative injury by reducing the production of reactive oxidative species (ROS), the translocation of NF-kB and activated protein 1, and the expression of intercellular adhesion molecule 1 (ICAM-1) [130]. In a study conducted in a population of 44 HD patients, daily supplementation with 455 mg of catechins extracted from green tea (amount equivalent of four cups of green tea/day) reduced HD-related ROS production, scavenging hydrogen peroxide, superoxide anion, and hypochlorous acid [131]. In HD patients, hypochlorous acid production promotes the atherogenic oxidation of LDL [132] and amplifies the hydrogen peroxide-induced vascular injury [133]. Since HD is not able to mechanically remove oxidized pro-atherosclerotic products, including oxidized LDL and phosphatidilcoline hydroperoxide, the use of powerful antioxidants that reduce the production of intradialytic ROS and protect against oxidative damage, such as green tea catechins, could help to slow down the progression of atherosclerotic vascular disease.

Caffeine is another widely studied compound of hot beverages, such as tea and coffee, with bioactive properties. Although its role in the development of hypertension and cardiovascular diseases (CVD) is still debated [134,135], there is evidence regarding its inverse association with type 2 diabetes [136]. However, the effect of coffee and tea consumption on renal function has been poorly investigated. The most recent observational study available found that daily coffee consumption of ≥1 cup/day significantly decreased the risk of CKD in a cohort of 8717 Korean subjects [137]. In a cohort of Japanese adults, the consumption of coffee, but not tea, was associated with increased eGFR [138]. The same findings have been reported in two more studies performed in Japan [139,140]. One study in Korean women found a protective role of coffee only in women with diabetes [141], while another Japanese study found no association at all [142]. A more recent study on a Western cohort has investigated the association between coffee and tea consumption and changes in the eGFR [143]. In this study coffee, but not tea, was found to be associated with a slightly higher eGFR among subjects of ≥46 years of age, and higher doses of coffee intake were observed to be associated with higher eGFRs. However, no associations with subsequent changes in eGFR or risk of rapid decline in eGFR were identified. These findings suggest that the increase in eGFR may not be related to the development of hyperfiltration, which is considered a risk factor for renal function, worsening over time because of its reflection on glomerular hypertension. On the other hand, experimental data suggest that caffeine has a negative influence on renal function in the presence of hypertension and pre-existing renal dysfunction, with an increase in proteinuria [144,145,146]. The mechanism related to this nephrotoxic effect of caffeine could be ascribed to its capacity to block renal adenosine receptors, which may augment angiotensin II-induced glomerular hypertension [146].

Hyperuricemia is a known risk factor for AKI and CKD progression [147]. An inverse association between coffee consumption and plasma uric acid was also described [148,149]. However, no association between total caffeine intake and hyperuricemia was found [149]. These findings suggest that components of coffee other than caffeine may contribute to the associations observed, since both caffeinated and decaffeinated coffee were found to be inversely associated with hyperuricemia. Since there is a strong direct correlation between insulin resistance and hyperuricemia [150,151,152,153], decreased insulin resistance and insulin levels associated with coffee consumption may lead to lower uric acid levels. Coffee is also the major source of the phenolic compound chlorogenic acid, a strong antioxidant that has been shown to reduce glycaemia [154,155]. It has also been speculated that non-caffeine xanthines in coffee may inhibit xanthine oxidase and reduce uric acid levels [148].

### 5.4. Nitrates and Nitrites 

Hypertension is a known risk factor for the development of CKD [156]. The BP-lowering effects of either a low-salt or a plant-based dietary approaches, such as the Dietary Approach to Stop Hypertension (DASH) diet, have been well assessed [11,12]. The DASH study demonstrates the efficacy of the synergy among plant matrices in achieving an overall hypotensive effect, making it difficult to discriminate the effect of an isolated dietary component [11]. An exception is represented by nitrates and nitrites, important molecules capable of independently affecting vascular function and regulate BP. They are notoriously referred to as carcinogenic when used as preservatives in processed meats, since this process leads to their conjugation with amino acids, with the release of toxic nitrosamines. However, it should be reported that less than 5% of the daily exposure to nitrates and nitrites comes from cured meat and that more than 85% of these molecules come from vegetables, particularly from some of the world’s healthiest roots and vegetables: beetroot and green leafy vegetables [157]. On the contrary, many observational studies are revealing that nitrates and nitrites are protective against CV risk [157], especially in the context of CKD [6]. This finding has recently led some authors to propose revising these compounds as actual dietary nutrients instead of mere additives, or even toxic substances [157].

**Figure 2:**
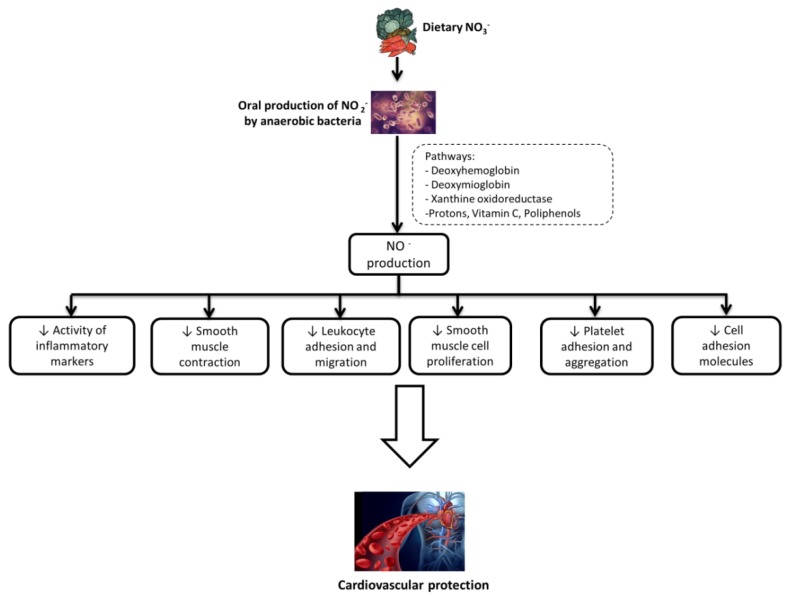
Mechanisms of cardioprotection by plant inorganic nitrates (NO_3_-).

The importance of dietary nitrates/nitrites for vascular health is traced to their action as precursors of nitric oxide (NO) via the enterosalivary pathway mediated by oral commensal bacteria [6] (Figure 2). In this context, oral microbiota dysbiosis could represent either a risk factor for endothelial dysfunction or a consequence of an imbalance in the microbiota-mediated nitrogen cycle [158]. 

NO is a fundamental modulator of endothelial function, vasodilatation, and BP. NO circulating levels are increased after nitrate and nitrite ingestion, either in the form of a supplement or diet-derived [158,159]. In addition, many experimental studies demonstrated the beneficial effects of nitrate and nitrite on BP [160,161,162,163,164], vascular compliance [163], endothelial function [163], and overall CV risk [165]. Moreover, the beneficial role of dietary nitrates/nitrites in improving physical performance has also been reported [157,160,162,164,165].

Nitrates and nitrites are fundamental in endogenous NO homeostasis and their insufficient dietary intake represents a risk factor for both the development and progression of endothelial dysfunction and CVD [157,158]. In addition, there is evidence that nitrate supplementation has a beneficial effect on ameliorating renal injury in animal models of hypertension and renal IR [166,167]. 

### 5.5. Alcohol

The adverse health effects caused by prolonged consumption of elevated amounts of alcohol and of acute alcohol intoxication are well known [168]. As to renal function, alcoholism is associated with a higher risk of glomerulonephritis [169,170], AKI [171], loss of renal function [172], and kidney graft failure [173]. However, moderate alcohol consumption has been related with health benefits, and its protective effects against CV mortality [174,175,176] and heart failure are well documented [177]. The beneficial effects of alcohol are dose- and gender-dependent, since women have less alcohol dehydrogenase than men [178]. A number of prospective observational studies have reported that moderate alcohol consumption could have a beneficial effect on the loss of renal function, also in patients with confirmed kidney disease [179,180,181,182,183,184]. In patients with IgA glomerulonephritis, proteinuria was found to be lower in light and moderate drinkers, while their creatinine clearance was higher as compared with abstainers and heavy drinkers [184]. Detrimental effects of alcohol abuse may include glomerular damage [185], hypertension, and hypertension-related nephrosclerosis [186,187]. On the other hand, the beneficial effects of alcohol in the kidney may be associated with its effects on BP, lipid profile (increased HDL), and vasoactive peptides regulation [180]. For instance, ethanol alters the activity of neurotransmitters and hormonal systems that affect the regulation of vasoactive substances, therefore affecting renal hemodynamics and function [180]. Additional mechanisms associating moderate alcohol intake with preserved renal function are: the reduced hyalinization of renal arterioles [188], the antioxidant activity of polyphenols contained in some alcoholic beverages (such as red wine), the increased activity of antioxidant enzymes [189], a lower lipid peroxidation, and a reduced protein oxidation [190]. 

## 6. Conclusions

In light of the above evidence, some general concepts need to be revised in patients with CKD and in healthy individuals, along with the presence of related/unrelated comorbidities. Cumulatively, a healthy lifestyle significantly decreases the risk of CKD. Raising people’s awareness of healthy lifestyles will help them to look after their own health by keeping modifiable risk factors under control. Maintaining an optimal weight, getting the right protein intake, and reducing calories and high-glycemic-index food are all important steps toward weight loss and metabolic rebalance. An excess of refined carbohydrates and a poor intake of high-biological-value protein are predisposing factors for weight gain and insulin resistance development. Awareness-raising measures to correct behavioral risk factors in the general population should be put in place in order to reduce CKD incidence. 

In conclusion, nutrition is increasingly being recognized as something far more important than a mere supply of dietary substances that provide raw materials and energy to the human body. It rather represents the most habitual factor functionally acting on the human body, and influencing its metabolism and immunity as a whole.

According to the Paracelsus principle “*sola dosis facit venenum*,” it is fundamental to establish the right dose of any substance for each individual and clinical condition. Nutrition is no exception to this principle: even for recognized health-promoting components, extrapolating rules that fit all situations in the general population could be misleading; such is the case for protein. In the future, the concept of personalized nutrition should be promoted even more. The nutrients, nutraceuticals, and xenobiotics discussed in this review confirm this: be they fiber, protein, FAs, curcumin, or alcohol, they can all be beneficial or deleterious depending on the level of intake and the clinical conditions of the individual, so these factors must always be accurately evaluated before designing any customized nutritional therapy and supplementation. 

## Figures and Tables

**Table 1:** Dietary protein recommendations for CKD patients stage 1–5 not on dialysis (adapted from [29]).

CKD Stage	Protein Intake Recommendation
Stage 1: renal damage with normal GFR (GFR > 90 mL/min/1.73m^2^)	Normal protein intake (RDA: 0.8 g/Kg/day)
Stage 2: slight reduction in renal function (GFR 60–89 mL/min/1.73m^2^)	Normal protein intake (RDA: 0.8 g/Kg/day)
Stage 3: moderate reduction of renal function (GFR 30–59 mL/min/1.73m^2^)	Protein restriction: 0.6–0.7 g/Kg/day)
Stage 4: severe reduction of renal function (GFR 15–29 mL/min/1.73m^2^)	Protein restriction: 0.6 g/Kg/day
Stage 5: end-stage renal disease (GFR < 15 mL/min/1.73m^2^)	Protein restriction: 0.3–0.4 g/Kg/day Supplementation with Keto acids required (1 tablet/5 Kg of body weight/day)

CKD: Chronic kidney disease; GFR: glomerular filtration rate; RDA: Recommended dietary allowances.

**Table 2:** Studies of omega-3 supplementation in CKD.

Study	Intervention	Results
Ferraro 2009	− Patients with IgA nephropathy were given 3 g of omega-3/day or renin-angiotensin system blockers alone	− Decreased proteinuria by 72.9% in the omega-3 group versus 11.3%; − No changes in GFR decline
Miller 2009	− Meta-analysis of 17 RCTs − Omega-3 supplementation (0.7–5.1 g/day)	− Decreased proteinuria was greater in the intervention groups − No differences in GFR decline
Hoogeven 2014	− CKD patients − 400 mg/day of EPA and DHA given together with margarine (equivalent of 2 portions of fatty fish/week) for 40 months	− The intervention was able to slow GFR decline
Bouzidi 2010	− CKD patients − Intervention group: 2.1 g/day of omega-3	− No differences in GFR between groups
Donadio 2001	− IgA nephropathy patients − 1.88 g/day of EPA + 1.47 g/day of DHA versus 3.76g of EPA and 2.94 g of DHA	− Both doses were equally effective in slowing increases in sCr levels, with more pronounced results in patients with moderate versus more advanced CKD
Alexopoulos 2004	− IgA Nephropathy patients − 0.85 g/day of EPA + 0.56 g/day of DHA versus standard treatment	− An increase in sCr of more than 50% was found in only 7% of patients in the intervention group versus 43% of patients in the control group

CKD: Chronic kidney disease; DHA: Docosahexaenoic acid; EPA: Eicosapentaenoic acid; GFR: Glomerular filtration rate; sCr: serum creatinine; RCTs: randomized clinical trials.

## References

[1] Jha V., Garcia-Garcia G., Iseki K., Li Z., Naicker S., Plattner B., Saran R., Wang A.Y., Yang C.W. (2013). Chronic kidney disease: Global dimension and perspectives. Lancet.

[2] Fouque D., Laville M. (2009). Low protein diets for chronic kidney disease in non-diabetic adults. Cochrane Database Syst. Rev..

[3] Cuenca-Sánchez M., Navas-Carrillo D., Orenes-Piñero E. (2015). Controversies surrounding high-protein diet intake: Satiating effect and kidney and bone health. Adv. Nutr..

[4] Slavin J. (2013). Fiber and prebiotics: Mechanisms and health benefits. Nutrients.

[5] Sabatino A., Regolisti G., Cosola C., Gesualdo L., Fiaccadori E. (2017). Intestinal Microbiota in Type 2 Diabetes and Chronic Kidney Disease. Curr. Database Rep..

[6] Cosola C., Rocchetti M.T., Cupisti A., Gesualdo L. (2018). Microbiota metabolites: Pivotal players of cardiovascular damage in chronic kidney disease. Pharmacol. Res..

[7] Mahmoodpoor F., Rahbar Saadat Y., Barzegari A., Ardalan M., Zununi Vahed S. (2017). The impact of gut microbiota on kidney function and pathogenesis. Biomed. Pharmacother..

[8] Ghosh S.S., Gehr T.W., Ghosh S. (2014). Curcumin and chronic kidney disease (CKD): Major mode of action through stimulating endogenous intestinal alkaline phosphatase. Molecules.

[9] De Angelis M., Montemurno E., Vannini L., Cosola C., Cavallo N., Gozzi G., Maranzano V., Di Cagno R., Gobbetti M., Gesualdo L. (2015). Effect of Whole-Grain Barley on the Human Fecal Microbiota and Metabolome. Appl. Environ. Microbiol..

[10] Fasano A. (2011). Zonulin and its regulation of intestinal barrier function: The biological door to inflammation, autoimmunity, and cancer. Physiol. Rev..

[11] Appel L.J., Moore T.J., Obarzanek E., Vollmer W.M., Svetkey L.P., Sacks F.M., Bray G.A., Vogt T.M., Cutler J.A., Windhauser M.M. (1997). A clinical trial of the effects of dietary patterns on blood pressure. DASH Collaborative Research Group. N. Engl. J. Med..

[12] Sacks F.M., Svetkey L.P., Vollmer W.M., Appel L.J., Bray G.A., Harsha D., Obarzanek E., Conlin P.R., Miller E.R., Simons-Morton D.G. (2001). Effects on blood pressure of reduced dietary sodium and the Dietary Approaches to Stop Hypertension (DASH) diet. DASH-Sodium Collaborative Research Group. N. Engl. J. Med..

[13] Aleixandre A., Miguel M. (2016). Dietary fiber and blood pressure control. Food Funct..

[14] Cosola C., De Angelis M., Rocchetti M.T., Montemurno E., Maranzano V., Dalfino G., Manno C., Zito A., Gesualdo M., Ciccone M.M. (2017). Beta-Glucans Supplementation Associates with Reduction in P-Cresyl Sulfate Levels and Improved Endothelial Vascular Reactivity in Healthy Individuals. PLoS ONE.

[15] Marchesi J.R., Adams D.H., Fava F., Hermes G.D., Hirschfield G.M., Hold G., Quraishi M.N., Kinross J., Smidt H., Tuohy K.M. (2016). The gut microbiota and host health: A new clinical frontier. Gut.

[16] Bliss D.Z., Stein T.P., Schleifer C.R., Settle R.G. (1996). Supplementation with gum arabic fiber increases fecal nitrogen excretion and lowers serum urea nitrogen concentration in chronic renal failure patients consuming a low-protein diet. Am. J. Clin. Nutr..

[17] Meijers B.K.I., De Preter V., Verbeke K., Vanrenterghem Y., Evenepoel P. (2010). p-Cresyl sulfate serum concentrations in haemodialysis patients are reduced by the prebiotic oligofructose-enriched inulin. Nephrol. Dial. Transplant..

[18] Salmean Y.A., Segal M.S., Langkamp-Henken B., Canales M.T., Zello G.A., Dahl W.J. (2013). Foods with added fiber lower serum creatinine levels in patients with chronic kidney disease. J. Ren. Nutr..

[19] Montemurno E., Cosola C., Dalfino G., Daidone G., De Angelis M., Gobbetti M., Gesualdo L. (2014). What would you like to eat, Mr CKD Microbiota? A Mediterranean Diet, please!. Kidney Blood Press Res..

[20] Cupisti A., D’Alessandro C., Gesualdo L., Cosola C., Gallieni M., Egidi M.F., Fusaro M. (2017). Non-Traditional Aspects of Renal Diets: Focus on Fiber, Alkali and Vitamin K1 Intake. Nutrients.

[21] Sumida K., Molnar M.Z., Potukuchi P.K., Thomas F., Lu J.L., Matsushita K., Yamagata K., Kalantar-Zadeh K., Kovesdy C.P. (2017). Constipation and incident CKD. J. Am. Soc. Nephrol..

[22] Cupisti A., Brunori G., Di Iorio B.R., D’Alessandro C., Pasticci F., Cosola C., Bellizzi V., Bolasco P., Capitanini A., Fantuzzi A.L. (2018). Nutritional treatment of advanced CKD: Twenty consensus statements. J. Nephrol..

[23] Rossi M., Johnson D.W., Xu H., Carrero J.J., Pascoe E., French C., Campbell K.L. (2015). Dietary protein-fiber ratio associates with circulating levels of indoxyl sulfate and p-cresyl sulfate in chronic kidney disease patients. Nutr. Metab. Cardiovasc. Dis..

[24] Esgalhado M., Kemp J.A., Damasceno N.R., Fouque D., Mafra D. (2017). Short-chain fatty acids: A link between prebiotics and microbiota in chronic kidney disease. Future Microbiol..

[25] Ramezani A., Massy Z.A., Meijers B., Evenepoel P., Vanholder R., Raj D.S. (2016). Role of the Gut Microbiome in Uremia: A Potential Therapeutic Target. Am. J. Kidney Dis..

[26] Scazzina F., Siebenhandl-Ehn S., Pellegrini N. (2013). The effect of dietary fibre on reducing the glycaemic index of bread. Br. J. Nutr..

[27] Slavin J.L. (2005). Dietary fiber and body weight. Nutrition.

[28] Yu K., Ke M.Y., Li W.H., Zhang S.Q., Fang X.C. (2014). The impact of soluble dietary fibre on gastric emptying, postprandial blood glucose and insulin in patients with type 2 diabetes. Asia Pac. J. Clin. Nutr..

[29] Aparicio M., Bellizzi V., Chauveau P., Cupisti A., Ecder T., Fouque D., Garneata L., Lin S., Mitch W.E., Teplan V. (2012). Keto acid therapy in predialysis chronic kidney disease patients: Final consensus. J. Ren. Nutr..

[30] Singh P., Germain M.J., Cohen L., Unruh M. (2014). The elderly patient on dialysis: Geriatric considerations. Nephrol. Dial. Transplant..

[31] Bernard S., Fouque D., Laville M., Zech P. (1996). Effects of low-protein diet supplemented with ketoacids on plasma lipids in adult chronic renal failure. Miner. Electrolyte MeTab..

[32] Malvy D., Maingourd C., Pengloan J., Bagros P., Nivet H. (1999). Effects of severe protein restriction with ketoanalogues in advanced renal failure. J. Am. Coll. Nutr..

[33] Cianciaruso B., Pota A., Pisani A., Torraca S., Annecchini R., Lombardi P., Capuano A., Nazzaro P., Bellizzi V., Sabbatini M. (2008). Metabolic effects of two low protein diets in chronic kidney disease stage 4–5—A randomized controlled trial. Nephrol. Dial. Transplant..

[34] Di Iorio B.R., Marzocco S., Bellasi A., De Simone E., Dal Piaz F., Rocchetti M.T., Cosola C., Di Micco L., Gesualdo L. (2018). Nutritional therapy reduces protein carbamylation through urea lowering in chronic kidney disease. Nephrol. Dial. Transplant..

[35] Di Iorio B.R., Minutolo R., De Nicola L., Bellizzi V., Catapano F., Iodice C., Rubino R., Conte G. (2003). Supplemented very low protein diet ameliorates responsiveness to erythropoietin in chronic renal failure. Kidney Int..

[36] Bellizzi V., Di Iorio B.R., De Nicola L., Minutolo R., Zamboli P., Trucillo P., Catapano F., Cristofano C., Scalfi L., Conte G., ERIKA Study-group (2007). Very low protein diet supplemented with ketoanalogs improves blood pressure control in chronic kidney disease. Kidney Int..

[37] Chauveau P., Combe C., Rigalleau V., Vendrely B., Aparicio M. (2007). Restricted protein diet is associated with decrease in proteinuria: Consequences on the progression of renal failure. J. Ren. Nutr..

[38] Di Iorio B.R., Bellizzi V., Bellasi A., Torraca S., D’Arrigo G., Tripepi G., Zoccali C. (2013). Phosphate attenuates the anti-proteinuric effect of very low-protein diet in CKD patients. Nephrol. Dial. Transplant..

[39] Chauveau P., Vendrely B., El Haggan W., Barthe N., Rigalleau V., Combe C., Aparicio M. (2003). Body composition of patients on a very low-protein diet: A two-year survey with DEXA. J. Ren. Nutr..

[40] Vendrely B., Chauveau P., Barthe N., El Haggan W., Castaing F., de Précigout V., Combe C., Aparicio M. (2003). Nutrition in hemodialysis patients previously on a supplemented very low protein diet. Kidney Int..

[41] Mitch W.E., Remuzzi G. (2004). Diets for patients with chronic kidney disease, still worth prescribing. J. Am. Soc. Nephrol..

[42] Brenner B.M., Meyer T.W., Hostetter T.H. (1982). Dietary protein intake and the progressive nature of kidney disease: The role of hemodynamically mediated glomerular injury in the pathogenesis of progressive glomerular sclerosis in aging, renal ablation, and intrinsic renal disease. N. Engl. J. Med..

[43] Knight E.L., Stampfer M.J., Hankinson S.E., Spiegelman D., Curhan G.C. (2003). The impact of protein intake on renal function decline in women with normal renal function or mild renal insufficiency. Ann. Intern. Med..

[44] Huang M.C., Chen M.E., Hung H.C., Chen H.C., Chang W.T., Lee C.H., Wu Y.Y., Chiang H.C., Hwang S.J. (2008). Inadequate energy and excess protein intakes may be associated with worsening renal function in chronic kidney disease. J. Ren. Nutr..

[45] Lawson J.A., Lazarus R., Kelly J.J. (2001). Prevalence and prognostic significance of malnutrition in chronic renal insufficiency. J. Ren. Nutr..

[46] Martin W.F., Armstrong L.E., Rodriguez N.R. (2005). Dietary protein intake and renal function. Nutr. Metab..

[47] Friedman A.N. (2004). High-protein diets: Potential effects on the kidney in renal health and disease. Am. J. Kidney Dis..

[48] Cirillo M., Lombardi C., Chiricone D., De Santo N.G., Zanchetti A., Bilancio G. (2014). Protein intake and kidney function in the middle-age population: Contrast between cross-sectional and longitudinal data. Nephrol. Dial. Transplant..

[49] Skov A.R., Toubro S., Bülow J., Krabbe K., Parving H.H., Astrup A. (1999). Changes in renal function during weight loss induced by high vs low-protein low-fat diets in overweight subjects. Int. J. Obes. Relat. Metab. Disord..

[50] Manninen A.H. (2005). High-protein diets are not hazardous for the healthy kidneys. Nephrol. Dial. Transplant..

[51] Marventano S., Kolacz P., Castellano S., Galvano F., Buscemi S., Mistretta A., Grosso G. (2015). A review of recent evidence in human studies of n-3 and n-6 PUFA intake on cardiovascular disease, cancer, and depressive disorders: Does the ratio really matter?. Int. J. Food Sci. Nutr..

[52] Guasch-Ferré M., Babio N., Martínez-González M.A., Corella D., Ros E., Martín-Peláez S., Estruch R., Arós F., Gómez-Gracia E., Fiol M. (2015). Dietary fat intake and risk of cardiovascular disease and all-cause mortality in a population at high risk of cardiovascular disease. Am. J. Clin. Nutr..

[53] Hammad S., Pu S., Jones P.J. (2016). Current Evidence Supporting the Link between Dietary Fatty Acids and Cardiovascular Disease. Lipids.

[54] Turolo S., Edefonti A., Syren M.L., Marangoni F., Morello W., Agostoni C., Montini G. (2018). Fatty Acids in Nephrotic Syndrome and Chronic Kidney Disease. J. Ren. Nutr..

[55] Das U.N., Mohan I.K., Raju T.R. (2001). Effect of corticosteroids and eicosapentaenoic acid/docosahexaenoic acid on pro-oxidant and anti-oxidant status and metabolism of essential fatty acids in patients with glomerular disorders. Prostaglandins Leukot. Essent. Fatty Acids.

[56] Fujita T., Nakamura N., Kumasaka R., Shimada M., Murakami R., Osawa H., Yamabe H., Okumura K. (2006). Comparison of lipid and fatty acid metabolism between minimal change nephrotic syndrome and membranous nephropathy. In Vivo.

[57] Aldámiz-Echevarría L., Vallo A., Aguirre M., Sanjurjo P., Gonzalez-Lamuño D., Elorz J., Prieto J.A., Andrade F., Rodríguez-Soriano J. (2007). Essential fatty acid deficiency profile in patients with nephrotic-range proteinuria. Pediatr. Nephrol..

[58] Oh J.S., Kim S.M., Sin Y.H., Kim J.K., Park Y., Bae H.R., Son Y.K., Nam H.K., Kang H.J., An W.S. (2012). Comparison of erythrocyte membrane fatty acid contents in renal transplant recipients and dialysis patients. Transplant. Proc..

[59] Sertoglu E., Kurt I., Tapan S., Uyanik M., Serdar M.A., Kayadibi H., El-Fawaeir S. (2014). Comparison of plasma and erythrocyte membrane fatty acid compositions in patients with end-stage renal disease and type 2 diabetes mellitus. Chem. Phys. Lipids.

[60] Sikorska-Wiśniewska M., Mika A., Śledziński T., Małgorzewicz S., Stepnowski P., Rutkowski B., Chmielewski M. (2017). Disorders of serum omega-3 fatty acid composition in dialyzed patients, and their associations with fat mass. Ren. Fail..

[61] Ghiggeri G.M., Ginevri F., Candiano G., Oleggini R., Perfumo F., Queirolo C., Gusmano R. (1987). Characterization of cationic albumin in minimal change nephropathy. Kidney Int..

[62] Ayala A., Muñoz M.F., Argüelles S. (2014). Lipid Peroxidation: Production, Metabolism, and Signaling Mechanisms of Malondialdehyde and 4-Hydroxy-2-Nonenal. Oxid. Med. Cell. Longev..

[63] Novak F., Borovska J., Vecka M., Rychlikova J., Vavrova L., Petraskova H., Zak A., Novakova O. (2017). Plasma Phospholipid Fatty Acid Profile is Altered in Both Septic and Non-Septic Critically Ill: A Correlation with Inflammatory Markers and Albumin. Lipids.

[64] Eid S., Abou-Kheir W., Sabra R., Daoud G., Jaffa A., Ziyadeh F.N., Roman L., Eid A.A. (2013). Involvement of renal cytochromes P450 and arachidonic acid metabolites in diabetic nephropathy. J. Biol. Regul. Homeost. Agents.

[65] Suzuki-Kemuriyama N., Matsuzaka T., Kuba M., Ohno H., Han S.I., Takeuchi Y., Isaka M., Kobayashi K., Iwasaki H., Yatoh S. (2016). Different Effects of Eicosapentaenoic and Docosahexaenoic Acids on Atherogenic High-Fat Diet-Induced Non-Alcoholic Fatty Liver Disease in Mice. PLoS ONE.

[66] Picklo M.J., Johnson L., Idso J. (2017). PPAR mRNA Levels Are Modified by Dietary n-3 Fatty Acid Restriction and Energy Restriction in the Brain and Liver of Growing Rats. J. Nutr..

[67] Clement L.C., Avila-Casado C., Macé C., Soria E., Bakker W.W., Kersten S., Chugh S.S. (2011). Podocyte-secreted angiopoietin-like-4 mediates proteinuria in glucocorticoid-sensitive nephrotic syndrome. Nat. Med..

[68] Shoji T., Kakiya R., Hayashi T., Tsujimoto Y., Sonoda M., Shima H., Mori K., Fukumoto S., Tahara H., Shioi A. (2013). Serum n-3 and n-6 polyunsaturated fatty acid profile as an independent predictor of cardiovascular events in hemodialysis patients. Am. J. Kidney Dis..

[69] Kuwamura Y., Shoji T., Okute Y., Yamazaki Y., Motoyama K., Morioka T., Mori K., Fukumoto S., Tsujimoto Y., Shioi A. (2018). Altered Serum n-6 Polyunsaturated Fatty Acid Profile and Risks of Mortality and Cardiovascular Events in a Cohort of Hemodialysis Patients. J. Ren. Nutr..

[70] Huang X., Stenvinkel P., Qureshi A.R., Risérus U., Cederholm T., Bárány P., Heimbürger O., Lindholm B., Carrero J.J. (2012). Essential polyunsaturated fatty acids, inflammation and mortality in dialysis patients. Nephrol. Dial. Transplant..

[71] Ferreri N.R., Hao S., Pedraza P.L., Escalante B., Vio C.P. (2012). Eicosanoids and tumor necrosis factor-alpha in the kidney. Prostaglandins Other Lipid Mediat..

[72] Zhang K., Wang J., Zhang H., Chen J., Zuo Z., Wang J., Huang H. (2013). Mechanisms of epoxyeicosatrienoic acids to improve cardiac remodeling in chronic renal failure disease. Eur. J. Pharmacol..

[73] Priante G., Musacchio E., Valvason C., Baggio B. (2009). EPA and DHA suppress AngII- and arachidonic acid-induced expression of profibrotic genes in human mesangial cells. J. Nephrol..

[74] Pavlov T.S., Ilatovskaya D.V., Levchenko V., Mattson D.L., Roman R.J., Staruschenko A. (2011). Effects of cytochrome P-450 metabolites of arachidonic acid on the epithelial sodium channel (ENaC). Am. J. Physiol. Ren. Physiol..

[75] Williams J.M., Sharma M., Anjaiahh S., Falck J.R., Roman R.J. (2007). Role of endogenous CYP450 metabolites of arachidonic acid in maintaining the glomerular protein permeability barrier. Am. J. Physiol. Ren. Physiol..

[76] Sharma M., McCarthy E.T., Reddy D.S., Patel P.K., Savin V.J., Medhora M., Falck J.R. (2009). 8,9-Epoxyeicosatrienoic acid protects the glomerular filtration barrier. Prostaglandins Other Lipid Mediat..

[77] Regner K.R., Zuk A., Van Why S.K., Shames B.D., Ryan R.P., Falck J.R., Manthati V.L., McMullen M.E., Ledbetter S.R., Roman R.J. (2009). Protective effect of 20-HETE analogues in experimental renal ischemia reperfusion injury. Kidney Int..

[78] Wong T.C., Chen Y.T., Wu P.Y., Chen T.W., Chen H.H., Chen T.H., Yang S.H. (2015). Ratio of Dietary n-6/n-3 Polyunsaturated Fatty Acids Independently Related to Muscle Mass Decline in Hemodialysis Patients. PLoS ONE.

[79] Choi A.M., Alam J. (1996). Heme oxygenase-1: Function, regulation, and implication of a novel stress-inducible protein in oxidant-induced lung injury. Am. J. Respir. Cell. Mol. Biol..

[80] Maines M.D. (1997). The heme oxygenase system: A regulator of second messenger gases. Annu. Rev. Pharmacol. Toxicol..

[81] Alam J., Cook J.L. (2003). Transcriptional regulation of the heme oxygenase-1 gene via the stress response element pathway. Curr. Pharm. Des..

[82] Abraham N.G., Kappas A. (2005). Heme oxygenase and the cardiovascular-renal system. Free Radic. Biol. Med..

[83] Ferraro P.M., Ferraccioli G.F., Gambaro G., Fulignati P., Costanzi S. (2009). Combined treatment with renin-angiotensin system blockers and polyunsaturated fatty acids in proteinuric IgA nephropathy: A randomized controlled trial. Nephrol. Dial. Transplant..

[84] Miller E.R., Juraschek S.P., Appel L.J., Madala M., Anderson C.A., Bleys J., Guallar E. (2009). The effect of n-3 long-chain polyunsaturated fatty acid supplementation on urine protein excretion and kidney function: Meta-analysis of clinical trials. Am. J. Clin. Nutr..

[85] Hoogeveen E.K., Geleijnse J.M., Kromhout D., Stijnen T., Gemen E.F., Kusters R., Giltay E.J. (2014). Effect of omega-3 fatty acids on kidney function after myocardial infarction: The Alpha Omega Trial. Clin. J. Am. Soc. Nephrol..

[86] Bouzidi N., Mekki K., Boukaddoum A., Dida N., Kaddous A., Bouchenak M. (2010). Effects of omega-3 polyunsaturated fatty-acid supplementation on redox status in chronic renal failure patients with dyslipidemia. J. Ren. Nutr..

[87] Donadio J.V., Larson T.S., Bergstralh E.J., Grande J.P. (2001). A randomized trial of high-dose compared with low-dose omega-3 fatty acids in severe IgA nephropathy. J. Am. Soc. Nephrol..

[88] Alexopoulos E., Stangou M., Pantzaki A., Kirmizis D., Memmos D. (2004). Treatment of severe IgA nephropathy with omega-3 fatty acids: The effect of a “very low dose” regimen. Ren. Fail..

[89] Syren M.L., Turolo S., Marangoni F., Milani G.P., Edefonti A., Montini G., Agostoni C. (2017). The polyunsaturated fatty acid balance in kidney health and disease: A review. Clin. Nutr..

[90] Metzler M., Pfeiffer E., Schulz S.I., Dempe J.S. (2013). Curcumin uptake and metabolism. Biofactors.

[91] Deng Q., Liang L., Liu Q., Duan W., Jiang Y., Zhang L. (2018). Autophagy is a major mechanism for the dual effects of curcumin on renal cell carcinoma cells. Eur. J. Pharmacol..

[92] Hernández-Reséndiz S., Correa F., García-Niño W.R., Buelna-Chontal M., Roldán F.J., Ramírez-Camacho I., Delgado-Toral C., Carbó R., Pedraza-Chaverrí J., Tapia E. (2015). Cardioprotection by curcumin post-treatment in rats with established chronic kidney disease. Cardiovasc. Drugs Ther..

[93] Jiménez-Osorio A.S., García-Niño W.R., González-Reyes S., Álvarez-Mejía A.E., Guerra-León S., Salazar-Segovia J., Falcón I., Montes de Oca-Solano H., Madero M., Pedraza-Chaverri J. (2016). The Effect of Dietary Supplementation with Curcumin on Redox Status and Nrf2 Activation in Patients with Nondiabetic or Diabetic Proteinuric Chronic Kidney Disease: A Pilot Study. J. Ren. Nutr..

[94] Fan Y., Chen H., Peng H., Huang F., Zhong J., Zhou J. (2017). Molecular Mechanisms of Curcumin Renoprotection in Experimental Acute Renal Injury. Front. Pharmacol..

[95] Shoskes D., Lapierre C., Cruz-Correa M., Muruve N., Rosario R., Fromkin B., Braun M., Copley J. (2005). Beneficial effects of the bioflavonoids curcumin and quercetin on early function in cadaveric renal transplantation: A randomized placebo controlled trial. Transplantation.

[96] Gupta S.C., Patchva S., Aggarwal B.B. (2013). Therapeutic roles of curcumin: Lessons learned from clinical trials. AAPS J..

[97] Aparicio-Trejo O.E., Tapia E., Molina-Jijón E., Medina-Campos O.N., Macías-Ruvalcaba N.A., León-Contreras J.C., Hernández-Pando R., García-Arroyo F.E., Cristóbal M., Sánchez-Lozada L.G. (2017). Curcumin prevents mitochondrial dynamics disturbances in early 5/6 nephrectomy: Relation to oxidative stress and mitochondrial bioenergetics. Biofactors.

[98] Ghosh S.S., Bie J., Wang J., Ghosh S. (2014). Oral supplementation with non-absorbable antibiotics or curcumin attenuates western diet-induced atherosclerosis and glucose intolerance in LDLR-/- mice—Role of intestinal permeability and macrophage activation. PLoS ONE.

[99] Abbate M., Zoja C., Remuzzi G. (2006). How does proteinuria cause progressive renal damage?. J. Am. Soc. Nephrol..

[100] Charalambous B.M., Stephens R.C., Feavers I.M., Montgomery H.E. (2007). Role of bacterial endotoxin in chronic heart failure: The gut of the matter. Shock.

[101] Sun P.P., Perianayagam M.C., Jaber B.L. (2009). Endotoxin-binding affinity of sevelamer: A potential novel anti-inflammatory mechanism. Kidney Int. Suppl..

[102] Bentala H., Verweij W.R., Huizinga-Van der Vlag A., van Loenen-Weemaes A.M., Meijer D.K., Poelstra K. (2002). Removal of phosphate from lipid A as a strategy to detoxify lipopolysaccharide. Shock.

[103] Liu W., Hu D., Huo H., Zhang W., Adiliaghdam F., Morrison S., Ramirez J.M., Gul S.S., Hamarneh R.S., Hodin A.R. (2016). Intestinal Alkaline Phosphatase Regulates Tight Junction Protein Levels. J. Am. Coll. Surg..

[104] Tada A., Takahashi K., Ishizuki K., Sugimoto N., Suematsu T., Arifuku K., Tahara M., Akiyama T., Ito Y., Yamazaki T. (2013). Absolute quantitation of stevioside and rebaudioside A in commercial standards by quantitative NMR. Chem. Pharm. Bull..

[105] Cardello H.M., Da Silva M.A., Damasio M.H. (1999). Measurement of the relative sweetness of stevia extract, aspartame and cyclamate/saccharin blend as compared to sucrose at different concentrations. Plant Foods Hum. Nutr..

[106] Ashwell M. (2015). Stevia, Nature’s Zero-Calorie Sustainable Sweetener: A New Player in the Fight against Obesity. Nutr. Today.

[107] Melis M.S. (1992). Stevioside effect on renal function of normal and hypertensive rats. J. Ethnopharmacol..

[108] Melis M.S. (1996). A crude extract of Stevia rebaudiana increases the renal plasma flow of normal and hypertensive rats. Braz. J. Med. Biol. Res..

[109] Shivanna N., Naika M., Khanum F., Kaul V.K. (2013). Antioxidant, anti-diabetic and renal protective properties of Stevia rebaudiana. J. Diabetes Complicat..

[110] Yuajit C., Chatsudthipong V. (2016). Nutraceutical for Autosomal Dominant Polycystic Kidney Disease Therapy. J. Med. Assoc. Thai..

[111] Yuajit C., Muanprasat C., Gallagher A.R., Fedeles S.V., Kittayaruksakul S., Homvisasevongsa S., Somlo S., Chatsudthipong V. (2014). Steviol retards renal cyst growth through reduction of CFTR expression and inhibition of epithelial cell proliferation in a mouse model of polycystic kidney disease. Biochem. Pharmacol..

[112] Yuajit C., Muanprasat C., Homvisasevongsa S., Chatsudthipong V. (2017). Steviol stabilizes polycystin 1 expression and promotes lysosomal degradation of CFTR and β-catenin proteins in renal epithelial cells. Biomed. Pharmacother..

[113] Noitem R., Yuajit C., Soodvilai S., Muanprasat C., Chatsudthipong V. (2018). Steviol slows renal cyst growth by reducing AQP2 expression and promoting AQP2 degradation. Biomed. Pharmacother..

[114] Lecumberri E., Dupertuis Y.M., Miralbell R., Pichard C. (2013). Green tea polyphenol epigallocatechin-3-gallate (EGCG) as adjuvant in cancer therapy. Clin. Nutr..

[115] Riegsecker S., Wiczynski D., Kaplan M.J., Ahmed S. (2013). Potential benefits of green tea polyphenol EGCG in the prevention and treatment of vascular inflammation in rheumatoid arthritis. Life Sci..

[116] Steinmann J., Buer J., Pietschmann T., Steinmann E. (2013). Anti-infective properties of epigallocatechin-3-gallate (EGCG), a component of green tea. Br. J. Pharmacol..

[117] Mukhtar H., Ahmad N. (1999). Mechanism of cancer chemopreventive activity of green Tea. Proc. Soc. Exp. Biol. Med..

[118] Baron J.A., Gerhardsson de Verdier M., Ekbom A. (1994). Coffee, tea, tobacco, and cancer of the large bowel. Cancer Epidemiol. Prev. Biomark..

[119] Gao Y.T., McLaughlin J.K., Blot W.J., Ji B.T., Dai Q., Fraumeni J.F. (1994). Reduced risk of esophageal cancer associated with green tea consumption. J. Natl. Cancer Inst..

[120] Sasazuki S., Tamakoshi A., Matsuo K., Ito H., Wakai K., Nagata C., Mizoue T., Tanaka K., Tsuji I., Inoue M. (2012). Green tea consumption and gastric cancer risk: An evaluation based on a systematic review of epidemiologic evidence among the Japanese population. Jpn. J. Clin. Oncol..

[121] Wang Y., Wang B., Du F., Su X., Sun G., Zhou G., Bian X., Liu N. (2015). Epigallocatechin-3-Gallate Attenuates Oxidative Stress and Inflammation in Obstructive Nephropathy via NF-κB and Nrf2/HO-1 Signalling Pathway Regulation. Basic Clin. Pharmacol. Toxicol..

[122] Wongmekiat O., Peerapanyasut W., Kobroob A. (2018). Catechin supplementation prevents kidney damage in rats repeatedly exposed to cadmium through mitochondrial protection. Naunyn Schmiedeberg Arch. Pharmacol..

[123] Yamabe N., Yokozawa T., Oya T., Kim M. (2006). Therapeutic potential of (-)-epigallocatechin 3-O-gallate on renal damage in diabetic nephropathy model rats. J. Pharmacol. Exp. Ther..

[124] Liang Y.J., Jian J.H., Liu Y.C., Juang S.J., Shyu K.G., Lai L.P., Wang B.W., Leu J.G. (2010). Advanced glycation end products-induced apoptosis attenuated by PPARdelta activation and epigallocatechin gallate through NF-kappaB pathway in human embryonic kidney cells and human mesangial cells. Diabetes Metab. Res. Rev..

[125] Cai S., Zhong Y., Li Y., Huang J., Zhang J., Luo G., Liu Z. (2013). Blockade of the formation of insoluble ubiquitinated protein aggregates by EGCG3”Me in the alloxan-induced diabetic kidney. PLoS ONE.

[126] Leu J.G., Lin C.Y., Jian J.H., Shih C.Y., Liang Y.J. (2013). Epigallocatechin-3-gallate combined with alpha lipoic acid attenuates high glucose-induced receptor for advanced glycation end products (RAGE) expression in human embryonic kidney cells. Anais Acad. Bras. Cienc..

[127] Sun W., Liu X., Zhang H., Song Y., Li T., Liu X., Liu Y., Guo L., Wang F., Yang T. (2017). Epigallocatechin gallate upregulates NRF2 to prevent diabetic nephropathy via disabling KEAP1. Free Radic. Biol. Med..

[128] Borges C.M., Papadimitriou A., Duarte D.A., Lopes de Faria J.M., Lopes de Faria J.B. (2016). The use of green tea polyphenols for treating residual albuminuria in diabetic nephropathy: A double-blind randomised clinical trial. Sci. Rep..

[129] Jialal I., Fuller C.J., Huet B.A. (1995). The effect of alpha-tocopherol supplementation on LDL oxidation. A dose-response study. Arterioscler. Thromb. Vasc. Biol..

[130] Chena W.C., Hayakawaa S., Shimizu K., Chien C.T., Lai M.K. (2004). Catechins prevents substance P-induced hyperactive bladder in rats via the downregulation of ICAM and ROS. Neurosci. Lett..

[131] Hsu S.P., Wu M.S., Yang C.C., Huang K.C., Liou S.Y., Hsu S.M., Chien C.T. (2007). Chronic green tea extract supplementation reduces hemodialysis-enhanced production of hydrogen peroxide and hypochlorous acid, atherosclerotic factors, and proinflammatory cytokines. Am. J. Clin. Nutr..

[132] Sutherland W.H., Walker R.J., de Jong S.A., van Rij A.M., Phillips V., Walker H.L. (1999). Reduced postprandial serum paraoxonase activity after a meal rich in used cooking fat. Arterioscler. Thromb. Vasc. Biol..

[133] Piotrowski J.J., Shah S., Alexander J.J. (1996). Mature human atherosclerotic plaque contains peroxidized phosphatidylcholine as a major lipid peroxide. Life Sci..

[134] Zhang Z., Hu G., Caballero B., Appel L., Chen L. (2011). Habitual coffee consumption and risk of hypertension: A systematic review and meta-analysis of prospective observational studies. Am. J. Clin. Nutr..

[135] Ding M., Bhupathiraju S.N., Satija A., van Dam R.M., Hu F.B. (2014). Long-term coffee consumption and risk of cardiovascular disease: A systematic review and a dose-response meta-analysis of prospective cohort studies. Circulation.

[136] Huxley R., Lee C.M., Barzi F., Timmermeister L., Czernichow S., Perkovic V., Grobbee D.E., Batty D., Woodward M. (2009). Coffee, decaffeinated coffee, and tea consumption in relation to incident type 2 diabetes mellitus: A systematic review with meta-analysis. Arch. Intern. Med..

[137] Jhee J.H., Nam K.H., An S.Y., Cha M.U., Lee M., Park S., Kim H., Yun H.R., Kee Y.K., Park J.T. (2018). Effects of Coffee Intake on Incident Chronic Kidney Disease: Community-Based Prospective Cohort Study. Am. J. Med..

[138] Saito M., Nemoto T., Tobimatsu S., Ebata M., Le Y., Nakajima K. (2011). Coffee consumption and cystatin-C-based estimated glomerular filtration rates in healthy young adults: Results of a clinical trial. J. Nutr. MeTab..

[139] Kotani K., Sakane N., Yamada T., Taniguchi N. (2010). Association between coffee consumption and the estimated glomerular filtration rate in the general Japanese population: Preliminary data regarding C-reactive protein concentrations. Clin. Chem. Lab. Med..

[140] Nakajima K., Hirose K., Ebata M., Morita K., Munakata H. (2010). Association between habitual coffee consumption and normal or increased estimated glomerular filtration rate in apparently healthy adults. Br. J. Nutr..

[141] Kim B.H., Park Y.S., Noh H.M., Sung J.S., Lee J.K. (2013). Association between Coffee Consumption and Renal Impairment in Korean Women with and without Diabetes: Analysis of the Fourth Korea National Health and Nutrition Examination Survey in 2008. Korean J. Fam. Med..

[142] Miyatake N., Shikata K., Makino H., Numata T. (2011). The relation between estimated glomerular filtration rate (eGFR) and coffee consumption in the Japanese. Health.

[143] Herber-Gast G.C., van Essen H., Verschuren W.M., Stehouwer C.D., Gansevoort R.T., Bakker S.J., Spijkerman A.M. (2016). Coffee and tea consumption in relation to estimated glomerular filtration rate: Results from the population-based longitudinal Doetinchem Cohort Study. Am. J. Clin. Nutr..

[144] Choi K.C., Lee J., Moon K.H., Park K.K., Kim S.W., Kim N.H. (1993). Chronic Caffeine Ingest ion Exacerbates 2-Kidney, 1-Clip Hypertension and Ameliorates Deoxycorticosterone Acetate-Salt Hypertension in Rats. Nephron.

[145] Kost C.K., Li P., Pfeifer C.A., Jackson E.K. (1994). Telemetric blood pressure monitoring in benign 2-kidney, 1-clip renovascular hypertension: Effect of chronic caffeine ingestion. J. Pharmacol. Exp. Ther..

[146] Tofovic S.P., Jackson E.K. (1999). Effects of long-term caffeine consumption on renal function in spontaneously hypertensive heart failure prone rats. J. Cardiovasc. Pharmacol..

[147] Fathallah-Shaykh S.A., Cramer M.T. (2014). Uric acid and the kidney. Uric acid and the kidney. Pediatr. Nephrol..

[148] Kiyohara C., Kono S., Honjo S., Todoroki I., Sakurai Y., Nishiwaki M., Hamada H., Nishikawa H., Koga H., Ogawa S. (1999). Inverse association between coffee drinking and serum uric acid concentrations in middle-aged Japanese males. Br. J. Nutr..

[149] Choi H.K., Curhan G. (2007). Coffee, tea, and caffeine consumption and serum uric acid level: The third national health and nutrition examination survey. Arthritis Rheum..

[150] Lee J., Sparrow D., Vokonas P.S., Landsberg L., Weiss S.T. (1995). Uric acid and coronary heart disease risk: Evidence for a role of uric acid in the obesity-insulin resistance syndrome. The Normative Aging Study. Am. J. Epidemiol..

[151] Rathmann W., Funkhouser E., Dyer A.R., Roseman J.M. (1998). Relations of hyperuricemia with the various components of the insulin resistance syndrome in young black and white adults: The CARDIA study. Coronary Artery Risk Development in Young Adults. Ann. Epidemiol..

[152] Emmerson B. (1998). Hyperlipidaemia in hyperuricaemia and gout. Ann. Rheum. Dis..

[153] Fam A.G. (2002). Gout, diet, and the insulin resistance syndrome. J. Rheumatol..

[154] Wu T., Giovannucci E., Pischon T., Hankinson S.E., Ma J., Rifai N., Rimm E.B. (2004). Fructose, glycemic load, and quantity and quality of carbohydrate in relation to plasma C-peptide concentrations in US women. Am. J. Clin. Nutr..

[155] Arion W.J., Canfield W.K., Ramos F.C., Schindler P.W., Burger H.J., Hemmerle H., Schubert G., Below P., Herling A.W. (1997). Chlorogenic acid and hydroxynitrobenzaldehyde: New inhibitors of hepatic glucose 6-phosphatase. Arch. Biochem. Biophys..

[156] McMahon G.M., Preis S.R., Hwang S.J., Fox C.S. (2014). Mid-adulthood risk factor profiles for CKD. J. Am. Soc. Nephrol..

[157] Bryan N.S., Ivy J.L. (2015). Inorganic nitrite and nitrate: Evidence to support consideration as dietary Nutrients. Nutr. Res..

[158] Briskey D., Tucker P.S., Johnson D.W., Coombes J.S. (2016). Microbiota and the nitrogen cycle: Implications in the development and progression of CVD and CKD. Nitric Oxide.

[159] Tsuchiya K., Kanematsu Y., Yoshizumi M., Ohnishi H., Kirima K., Izawa Y., Shikishima M., Ishida T., Kondo S., Kagami S. (2005). Nitrite is an alternative source of NO in vivo. Am. J. Physiol. Heart Circ. Physiol..

[160] Vanhatalo A., Bailey S.J., Blackwell J.R., DiMenna F.J., Pavey T.G., Wilkerson D.P., Benjamin N., Winyard P.G., Jones A.M. (2010). Acute and chronic effects of dietary nitrate supplementation on blood pressure and the physiological responses to moderate-intensity and incremental exercise. Am. J. Physiol. Regul. Integr. Comp. Physiol..

[161] Greenway F.L., Predmore B.L., Flanagan D.R., Giordano T., Qiu Y., Brandon A., Lefer D.J., Patel R.P., Kevil C.G. (2012). Single-dose pharmacokinetics of different oral sodium nitrite formulations in diabetes patients. Diabetes Technol. Ther..

[162] Kelly J., Fulford J., Vanhatalo A., Blackwell J.R., French O., Bailey S.J., Gilchrist M., Winyard P.G., Jones A.M. (2013). Effects of short-term dietary nitrate supplementation on blood pressure, O_2_ uptake kinetics, and muscle and cognitive function in older adults. Am. J. Physiol. Regul. Integr. Comp. Physiol..

[163] Houston M., Hays L. (2014). Acute effects of an oral nitric oxide supplement on blood pressure, endothelial function, and vascular compliance in hypertensive patients. J. Clin. Hypertens.

[164] Biswas O.S., Gonzalez V.R., Schwarz E.R. (2015). Effects of an oral nitric oxide supplement on functional capacity and blood pressure in adults with prehypertension. J. Cardiovasc. Pharmacol. Ther..

[165] Lansley K.E., Winyard P.G., Fulford J., Vanhatalo A., Bailey S.J., Blackwell J.R., DiMenna F.J., Gilchrist M., Benjamin N., Jones A.M. (2011). Dietary nitrate supplementation reduces the O_2_ cost of walking and running: A placebo-controlled study. J. Appl. Physiol..

[166] Tripatara P., Patel N.S., Webb A., Rathod K., Lecomte F.M., Mazzon E., Cuzzocrea S., Yaqoob M.M., Ahluwalia A., Thiemermann C. (2007). Nitrite-derived nitric oxide protects the rat kidney against ischemia/reperfusion injury in vivo: Role for xanthine oxidoreductase. J. Am. Soc. Nephrol..

[167] Tsuchiya K., Tomita S., Ishizawa K., Abe S., Ikeda Y., Kihira Y., Tamaki T. (2010). Dietary nitrite ameliorates renal injury in L-NAME-induced hypertensive rats. Nitric Oxide.

[168] National Institute of Alcohol Abuse and Alcoholism (2000). 10th Special Report on the US Congress on Alcohol and Health.

[169] Keller C.K., Andrassy K., Waldherr R., Ritz E. (1994). Postinfectious glomerulonephritis—Is there a link to alcoholism?. Q. J. Med..

[170] Nasr S.H., Markowitz G.S., Stokes M.B., Said S.M., Valeri A.M., D’Agati V.D. (2008). Acute postinfectious lomerulonephritis in the modern era: Experience with 86 adults and review of the literature. Medicine.

[171] Bagshaw S.M., Laupland K.B., Doig C.J., Mortis G., Fick G.H., Mucenski M., Godinez-Luna T., Svenson L.W., Rosenal T. (2005). Prognosis for long-term survival and renal recovery in critically ill patients with severe acute renal failure: A population-based study. Crit. Care.

[172] Shankar A., Klein R., Klein B.E. (2006). The association among smoking, heavy drinking, and chronic kidney disease. Am. J. Epidemiol..

[173] Gueye A.S., Chelamcharla M., Baird B.C., Nguyen C., Tang H., Barenbaum A.L., Koford J.K., Shihab F., Goldfarb-Rumyantzev A.S. (2007). The association between recipient alcohol dependency and long-term graft and recipient survival. Nephrol. Dial. Transplant..

[174] Thun M.J., Peto R., Lopez A.D., Monaco J.H., Henley S.J., Heath C.W., Doll R. (1997). Alcohol consumption and mortality among middle-aged and elderly, U.S. adults. N. Engl. J. Med..

[175] Mukamal K.J., Maclure M., Muller J.E., Sherwood J.B., Mittleman M.A. (2001). Prior alcohol consumption and mortality following acute myocardial infarction. JAMA.

[176] Rimm E.B., Stampfer M.J. (2002). Wine, beer, and spirits: Are they really horses of a different color?. Circulation.

[177] Bryson C.L., Mukamal K.J., Mittleman M.A., Fried L.P., Hirsch C.H., Kitzman D.W., Siscovick D.S. (2006). The association of alcohol consumption and incident heart failure: The Cardiovascular Health Study. J. Am. Coll. Cardiol..

[178] Kloner R.A., Rezkalla S.H. (2007). To drink or not to drink? That is the question. Circulation.

[179] Knight E.L., Stampfer M.J., Rimm E.B., Hankinson S.E., Curhan G.C. (2003). Moderate alcohol intake and renal function decline in women: A prospective study. Nephrol. Dial. Transplant..

[180] Chung F.M., Yang Y.H., Shieh T.Y., Shin S.J., Tsai J.C., Lee Y.J. (2005). Effect of alcohol consumption on estimated glomerular filtration rate and creatinine clearance rate. Nephrol. Dial. Transplant..

[181] White S.L., Polkinghorne K.R., Cass A., Shaw J.E., Atkins R.C., Chadban S.J. (2009). Alcohol consumption and 5-year onset of chronic kidney disease: The AusDiab study. Nephrol. Dial. Transplant..

[182] Schaeffner E.S., Kurth T., de Jong P.E., Glynn R.J., Buring J.E., Gaziano J.M. (2005). Alcohol consumption and the risk of renal dysfunction in apparently healthy men. Arch. Intern. Med..

[183] Reynolds K., Gu D., Chen J., Tang X., Yau C.L., Yu L., Chen C.S., Wu X., Hamm L.L., He J. (2008). Alcohol consumption and the risk of end-stage renal disease among Chinese men. Kidney Int..

[184] Kaartinen K., Niemela O., Syrjanen J., Porsti I., Harmoinen A., Pasternack A., Huhtala H., Mustonen J. (2009). Alcohol consumption and kidney function in IgA glomerulonephritis. Nephron Clin. Pract..

[185] Amore A., Coppo R., Roccatello D., Piccoli G., Mazzucco G., Gomez-Chiarri M., Lamm M.E., Emancipator S.N. (1994). Experimental IgA nephropathy secondary to hepatocellular injury induced by dietary deficiencies and heavy alcohol intake. Lab. Investig..

[186] Corrao G., Bagnardi V., Zambon A., Arico S. (1999). Exploring the dose-response relationship between alcohol consumption and the risk of several alcohol-related conditions: A meta-analysis. Addiction.

[187] Whelton P.K., Perneger T.V., He J., Klag M.J. (1996). The role of blood pressure as a risk factor for renal disease: A review of the epidemiologic evidence. J. Hum. Hypertens..

[188] Burchfiel C.M., Tracy R.E., Chyou P.H., Strong J.P. (1997). Cardiovascular risk factors and hyalinization of renal arterioles at autopsy. The Honolulu Heart Program. Arterioscler. Thromb. Vasc. Biol..

[189] Rodrigo R., Miranda A., Vergara L. (2011). Modulation of endogenous antioxidant system by wine polyphenols in human disease. Clin. Chim. Acta.

[190] Schaeffner E., Ritz E. (2012). Alcohol and kidney damage: A Janus-faced relationship. Kidney Int..

